# Time barrier to trade: Data on 190 economies’ export and import time, 2005–2018

**DOI:** 10.1016/j.dib.2018.11.054

**Published:** 2018-11-14

**Authors:** Wenchao Li

**Affiliations:** National University of Singapore, Singapore

## Abstract

This data article presents data on time to export and import across 190 economies, for the years 2005–2018. The data can foster research on international trade, and are of great academic and political value given the growing awareness and importance of time as a trade barrier. The data are publicly available at https://www.doingbusiness.org/data. A subset of the data is used in the related research data article, “Time barrier to export for OECD countries” (Li, 2018). Data on the number of documents required in these economies to export and import are also presented, for the years 2005–2015.

**Specifications table**

**Table:** 

Subject area	*Economics*
More specific subject area	*International trade, time barrier*
Type of data	*Table, figures, .xlsx file*
How data were acquired	*From database provided by the World Bank*
Data format	*Raw, partially analyzed*
Experimental factors	*Downloadable from the website*http://www.doingbusiness.org/data
Experimental features	*Panel data*
Data source location	*Institution: The World Bank*
Data accessibility	*With this article and publicly available at:*http://www.doingbusiness.org/data
Related research article	Li, Wenchao, “Time barrier to export for OECD countries,” *Economics Letters*, forthcoming, [1].

**Value of the data**

• The data are designed to be used by researchers who are interested in an important source of trade costs: time delays due to border and documentary compliance.
• The dataset provides rich information on export and import time across 190 economies, dating back to 2005.
• The data facilitate future work on whether time delays affect trading patterns of different regions or different groups of countries (developing or developed, high- or low-income) differently, how trade policies and reforms boost trade, which of export and import time plays a more important role in bilateral trade, etc.

## 1 Data

**Fig. 1:**
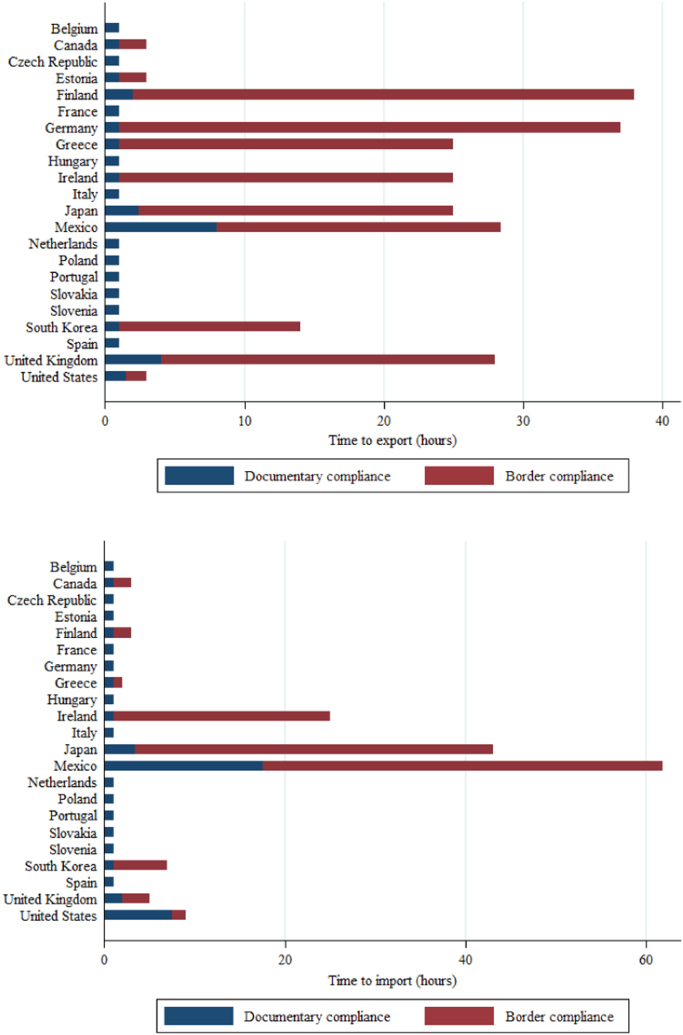
Time to export and import for 22 OECD countries, most recent.

**Fig. 2:**
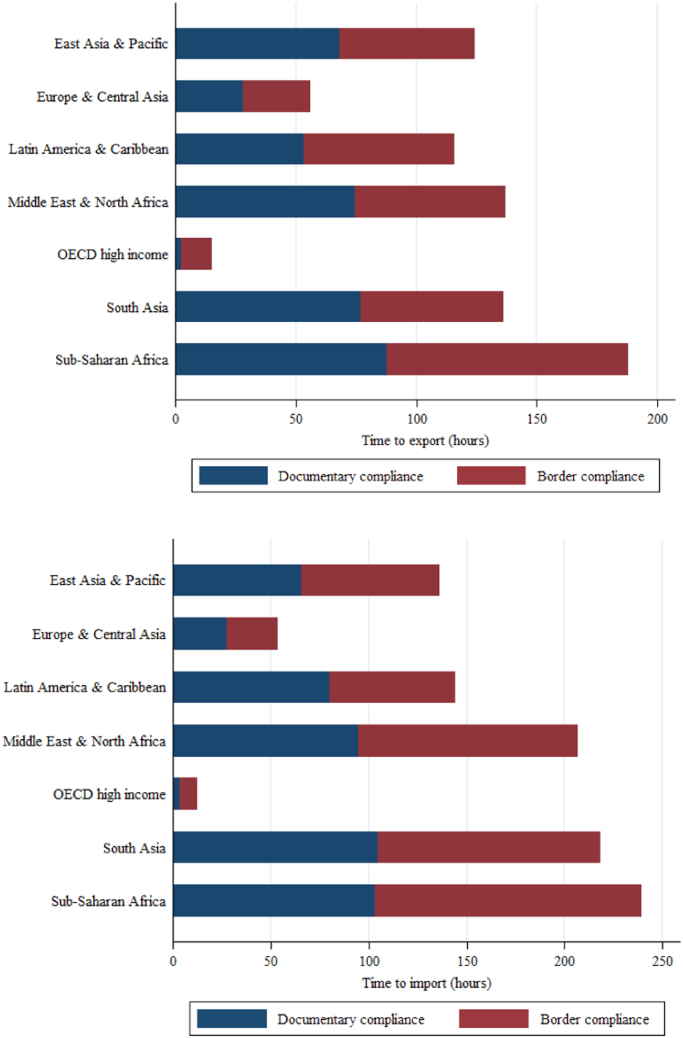
Time to export and import for different regions, most recent.

**Fig. 3:**
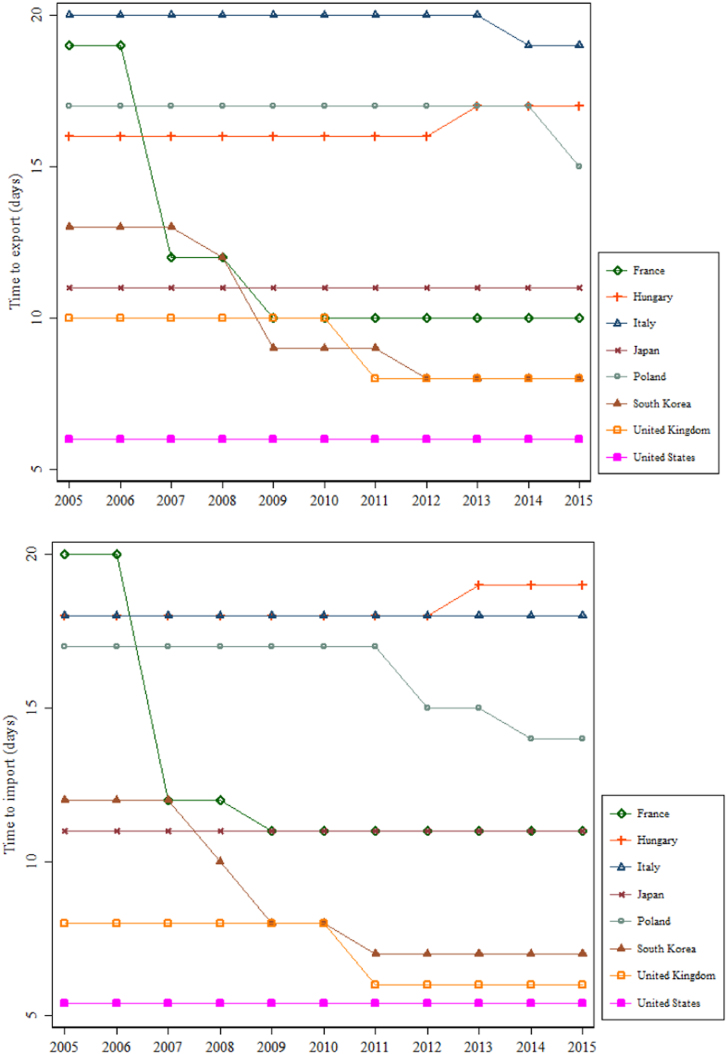
Trend in time to export and import for selected OECD countries, 2005–2015.

This data article describes data on export and import time—an important form of trade barrier in contemporary international trade [2]. From 2005, the World Bank Doing Business measures the time and cost to export and import every year. It records the time associated with three sets of procedures—documentary compliance, border compliance, and domestic transport—within the overall logistical process of exporting or importing a shipment of goods. Table 1 describes these procedures. The data cover 190 economies, for the years 2005–2018. Fig. 1 reports the time to export and import for 22 OECD countries—the US, Canada, Japan, Korea, Mexico, and 17 EU member countries. Fig. 2 reports the time for seven regions in the world—East Asia and Pacific, Europe and Central Asia, Latin America and Caribbean, Middle East and North Africa, OECD high income, South Asia, and Sub-Saharan Africa. In these two figures, the documentary compliance time and the border compliance time are presented. Fig. 3 reports the trends in days to export and import for eight OECD countries—France, Hungary, Italy, Japan, Poland, South Korea, the UK, and the US—over the period 2005–2015.

**Table 1:** Three sets of procedures in the process of exporting and importing.

**Documentary compliance**	

Compliance with documentary requirements of government agencies of the origin economy, the destination economy, and any transit economies	
Time	Obtaining, preparing, processing, presenting, and submitting documents
	All mandatory electronic or paper submissions of information requested
**Border compliance**	

Compliance with the economy׳s customs regulations and regulations relating to other inspections	
Time	Customs clearance and inspections
	Inspections by agencies other than customs (if take place in more than 20% of cases)
	Handling at the port or border and at other locations

**Domestic transport**	

Transporting the shipment from a warehouse in the economy׳s largest business city to its most widely used seaport or land border	
Time	Actual transport, any traffic delays and road police checks
	Loading or unloading at the warehouse or border
	Covering all transport paths

**Fig. 4:**
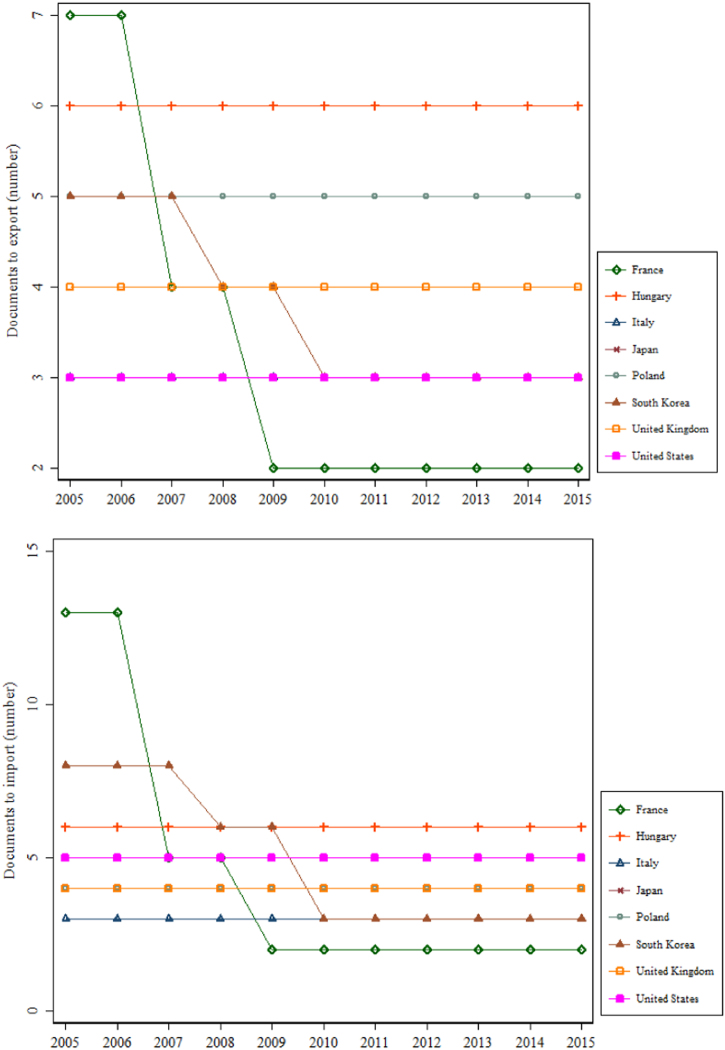
Trend in the number of documents to export and import for selected OECD countries, 2005–2015.

This data article also describes data on the number of documents required to export and import, for the years 2005–2015. Fig. 4 reports the trends in the number of documents for the eight OECD countries as in Fig. 3.

The dataset discussed in this data article is attached as a supplementary material, “Export_import_all.xlsx”, and also publicly available at http://www.doingbusiness.org/data. The related research data article, “Time barrier to export for OECD countries” [1], is based on the data for 22 OECD countries.^1^

## 2 Experimental design, materials, and methods

The data on 190 economies’ time to export and import presented in this article are collected by Doing Business, the World Bank, through a questionnaire administered to local freight forwarders, customs brokers, port authorities, and traders. The methodology used to construct the dataset was initially developed by Djankov et al. [4]. It has been revised for improvement and expansion in Doing Business 2015.

Below, the author first present the assumptions that are made in the case studies of Doing Business with the aim of making the data internationally comparable. Then, the author describes how the components of the recent data on export and import time are measured.

### 2.1 Assumptions

The following assumptions about the traded goods and the transactions are made in the case studies of Doing Business, which are essential to make the data comparable across economies.

• A shipment is a unit of trade. A shipment in a warehouse in the largest business city of the exporter travels to a warehouse in the largest business city of the importer. For 11 economies, data are also available for the second largest business city.
• Each economy exports the product with the largest export value to the economy that is the largest purchaser of this product. Each economy imports a standardized shipment of 15 metric tons of containerized auto parts from the economy from which it imports the largest value of auto parts.
• Export shipments do not necessarily need to be containerized, while import shipments do. When containerized, a shipment consists of cargo belonging to a single Harmonized System (HS) classification code.
• The traded product is new.
• The mode and route of transport are the most widely used ones for the selected product-partner pair.

### 2.2 Procedures in the process of exporting and importing

In June 2017, Doing Business completed the most recent round of collection of data on time to export and import. The time is measured in hours. If the process takes several days, one day is converted to 24 h (for example, 22 days are 528 h). Doing Business separately records the time associated with three sets of procedures—documentary compliance, border compliance, and domestic transport. See Table 1.

Documentary compliance refers to compliance with the documentary requirements of all government agencies of the origin economy, the destination economy, and any transit economies. The time for this segment includes the time for obtaining, preparing, processing, presenting, and submitting documents. Considered are all mandatory electronic or paper submissions of information requested by any government agencies that are relevant to the shipment—such as customs, port authorities, road police, border guards, standardization agencies, ministries or departments of agriculture or industry, national security agencies, central banks, etc.

Border compliance refers to compliance with the economy׳s customs regulations and with regulations relating to other inspections that are mandatory in order for the shipment to cross the economy׳s border. The time for this segment includes the time for customs clearance and inspections. The time for inspections by agencies other than customs is also included if such inspections take place in more than 20% of cases. Both the time for handling that takes place at the port or border and the time at other locations are included. See Figs. 1 and 2. Also note that in trade between members of the European Union (EU) and other customs unions, the border compliance time is negligible.

Domestic transport refers to transporting the shipment from a warehouse in the economy׳s largest business city to its most widely used seaport or land border. The time for this segment includes the time for the actual transport, any traffic delays and road police checks, as well as time spent on loading or unloading at the warehouse or border. In cases where the shipment travels from the warehouse to a customs post or terminal for clearance or inspections and then travels onward to the port or border, the time for both transport paths is included. The domestic transport time catches relatively less attention of researchers and policymakers and is therefore not presented in the figures, as this time segment is affected by many external factors like the geography and topography, road capacity and general infrastructure, proximity to the port or border, and the location of warehouses.

Before Doing Business revised the methodology used to construct its dataset in 2015, the time to export and import was measured in days. The total time associated with four sets of procedures—document preparation, customs clearance and inspections, inland transport and handling, and port and terminal handling—was recorded. The numbers of documents to export and import for each economy were also recorded. See Figs. 3 and 4. Nordås et al. [5] provide an example of using the number of signatures needed for exporters as an instrumental variable for export time. They show that this method gives similar parameter estimates with a stronger statistical significance, compared with directly using export time. As noted earlier, from 2015 onwards, Doing Business measures the time to export and import in hours.

## Footnotes

1 Costinot et al. [3] also make use of these data.
