# Supplementary material for: Time barrier to trade: Data on 190 economies’ export and import time, 2005–2018
Source: Data Brief. 2018 Nov 14;22:508–15. doi: 10.1016/j.dib.2018.11.054 (PMC6321974; doi:10.1016/j.dib.2018.11.054)
Supplement: Supplementary file 1 — Supplementary material [file mmc1.pdf]

Declaration

The author declares that there is no conflict of interest.

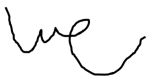

Wenchao Li
